# Comment on “A Biochemical Evaluation on Inflammatory Markers after Ureteroscopic Lithotripsy”

**DOI:** 10.1155/2014/919801

**Published:** 2014-09-10

**Authors:** Petros Sountoulides, Athanasios Bantis

**Affiliations:** ^1^Urology Department, General Hospital of Veria, 55133 Thessaloniki, Greece; ^2^Urology Department, University Hospital of Alexandroupolis, Dragana, 68100 Alexandroupolis, Greece

 Endoscopic lithotripsy for upper urinary tract stones is the cornerstone of treatment and a routine procedure for practicing urologists. Although highly effective in reducing or eliminating stone burden, ureteroscopy is associated with a significant risk of infectious complications, namely, fever, pyelonephritis, and rarely sepsis [1].

Although prophylactic administration of antibiotics is recommended in ureteroscopic lithotripsy cases in order to ensure sterile urine [2], infectious complications following ureteroscopic lithotripsy do not seem to be always related to the presence of bacteria in the urine. Infectious complications following URS lithotripsy are associated with maneuvers in the upper urinary tract and even related to noninfectious factors such as aseptic renal inflammation [3].

In this concept we tried to identify “markers” of inflammation (serum IL-6 and TNF-*α*) that would potentially aid in identifying those patients with urinary stones that are more prone to the postoperative development of infectious complications.

Aydin et al. in their letter to the editor commented that urolithiasis causes elevation of IL-6 through two distinct mechanisms: (a) a mechanical one as stones cause direct damage to the endothelium and (b) in response to smooth contraction during renal or ureteral colic [4].

We thank Aydin et al. for their insightful comments. However from a clinical standpoint one can challenge this suggestion. First, all patients in our study were scheduled for standard URS lithotripsy in an elective basis and not as emergency procedures. Standard urological practice for patients under acute renal colic is to alleviate the pain with anti-inflammatory or opioid drugs and to relieve the obstructed upper urinary tract by means of stenting or placing a nephrostomy. Therefore the factor of IL-6 elevation caused by muscle spasm and pain does not apply to our study [5]. Moreover the pain caused by a stone in the ureter is attributed to the acute distention of the renal capsule caused by the obstruction combined with the increased peristalsis of the smooth muscle layer of the ureter. Therefore the paper cited by Aydin et al. (Brandt et al., “The role of exercise-induced myokines in muscle homeostasis and the defense against chronic diseases,” 2010) is irrelevant to renal colic and does not apply to our study findings. Second, since damage to the urothelium is a standard event when a stone is present in the ureter how do Aydin et al. account for the fact that IL-6 was not elevated in all patients with stones prior to intervention?

The issue of identifying patients at risk of infectious complications following ureteroscopy for stones will raise significant concern in the future, and we consider that looking at markers such as IL-6 and TNF might help in shedding light on this area.
